# Kinship composition in mammals

**DOI:** 10.1098/rsos.230486

**Published:** 2023-07-19

**Authors:** André S. Pereira, Delphine De Moor, Catarina Casanova, Lauren J. N. Brent

**Affiliations:** ^1^ Centre for Research in Animal Behavior, University of Exeter, Exeter EX4 4QG, UK; ^2^ Research Centre for Anthropology and Health, Department of Life Sciences, University of Coimbra, 3000-456 Coimbra, Portugal; ^3^ CAPP, ISCSP, University of Lisbon, 1300-663 Lisbon, Portugal

**Keywords:** relatedness, group-living, cooperation, sociality, pedigree, kinship

## Abstract

Understanding the evolution of group-living and cooperation requires information on who animals live and cooperate with. Animals can live with kin, non-kin or both, and kinship structure can influence the benefits and costs of group-living and the evolution of within-group cooperation. One aspect of kinship structure is kinship composition, i.e. a group-level attribute of the presence of kin and/or non-kin dyads in groups. Despite its putative importance, the kinship composition of mammalian groups has yet to be characterized. Here, we use the published literature to build an initial kinship composition dataset in mammals, laying the groundwork for future work in the field. In roughly half of the 18 species in our sample, individuals lived solely with same-sex kin, and, in the other half, individuals lived with related and unrelated individuals of the same sex. These initial results suggest that it is not rare for social mammals to live with unrelated individuals of the same sex, highlighting the importance of considering indirect and direct fitness benefits as co-drivers of the evolution of sociality. We hope that our initial dataset and insights will spur the study of kinship structure and sociality towards new exciting avenues.

## 1. Introduction

Group-living, wherein multiple animals live in stable social groups, is widespread across taxa [1]. Group-living is favoured by natural selection when its benefits to individuals exceed its costs [1]. Characterizing the cost-benefit trade-off of living in groups thus constitutes an important step towards understanding when and why group-living evolves [2]. The trade-off between the costs and benefits of group-living is deeply intertwined with cooperation. The benefits accrued from cooperative interactions can drive the evolution of living in groups, for example in cooperative breeders. Alternatively, when factors such as predation drive group-living, intra-group cooperation can offset some of the costs and enhance some of the benefits of living in groups [3]. Understanding cooperation's role in the evolution of group-living and how group-living promotes the evolution of cooperation remain major challenges in behavioural ecology [4].

A key factor mediating the evolution of group-living and within-group cooperation is the kinship structure of groups, i.e. how group members are related to each other. Kinship structure impacts the type of benefits groupmates gain from living and interacting with each other via indirect fitness [5], whereby individuals benefit by helping their close relatives, who can pass on shared genes to the next generation. A classic example of this scenario is cooperative breeding societies, wherein the indirect fitness benefits gained by helping related dominant individuals are potentially higher than the direct fitness benefits gained by attempting to breed elsewhere. More generally, all else being equal, cooperating with kin instead of non-kin can provide larger inclusive fitness benefits (i.e. the sum of direct and indirect benefits). This is because individuals gain not only direct fitness benefits from such cooperation, but also indirect fitness benefits from increasing their kin's survival or reproduction through, for example, social thermoregulation, sharing food and information, providing social tolerance and coalitionary support [6]. Indeed, affiliative, i.e. positive non-aggressive non-reproductive, interactions between related groupmates are generally associated with higher individual fitness [6–8].

Indirect fitness benefits obtained by cooperating with kin have therefore long been considered crucial for the evolution of group-living and cooperation. As such, groups featuring unrelated members of the same sex have been considered to be more difficult to explain and have been generally thought to be rarer [9,10]. Yet, societies often feature non-kin. For example, previous research characterized the kinship between group members of cooperative breeding birds and found, contrary to predictions, that at least 45% of species included in the study regularly lived with non-kin [10]. Non-kin featured as non-breeding helpers in pair-nesting units in 40 of 158 species examined, and as breeders in all 55 units in which reproduction and parental care were shared [10]. These results suggest that cooperative breeding might have evolved via previously unconsidered routes [10] and highlight the importance of characterizing the kinship structure of groups to understand the drivers of the evolution of group-living [4]. Yet, despite the relatively large body of research on cooperation and kin biases in social behaviour in mammals [6,9], a categorization of the kinship composition of mammalian societies is missing.

Kinship composition, i.e. a group-level attribute of the presence of kin and/or non-kin dyads in groups, is a component of kinship structure that complements the better-characterized mean relatedness [11–14]. Kinship composition differs from mean relatedness by providing dyadic level information. Mean relatedness is calculated by summing the coefficient of relatedness of dyads in a group and dividing it by the number of dyads. Mean relatedness is often calculated from purely genetic data, such as molecular markers, and at the level of the group, i.e. including all group members for which data are available [15,16]. Mean relatedness is a measure of the central tendency of dyadic kinship, so it cannot be explicit about whether groups constitute only kin dyads, non-kin dyads or a mixture of both. Groups with high mean relatedness can still contain non-kin, and those with low mean relatedness can still contain kin. For example, two groups of three individuals with mean relatedness of 0.125 can have different kinship compositions. One of the groups could be fully related, containing 100% of kin dyads, for example three maternal first cousins, i.e. three individuals who had the same maternal grandparents (*r* = 0.125 between all pairs of individuals in the group). The other group could feature an individual, its paternal half-sibling and a maternal first cousin, who in turn are unrelated to each other. As such, this group would feature a mix of kin and non-kin dyads (containing 67% of kin dyads on a continuous scale) but would also have a mean relatedness of 0.125 (0.25+0.125+0=0.375/3=0.125). By complementing mean relatedness with data on kinship composition we can characterize the types of dyadic kinship categories present in groups. This allows us to investigate how within-group cooperation is patterned between kin and non-kin dyads, and helps us better understand how groups are formed and maintained.

Here, we aim to lay the foundation for research on the kinship composition of mammals by providing an initial overview of its taxonomic representation. To do this, we propose definitions of key concepts and build an initial dataset of the kinship composition of mammals. We quantified same-sex kinship composition by determining whether same-sex group members featured kin, non-kin or both, and respectively classed groups as related, unrelated or mix-related. Both kinship structure and social structure have core dyadic components. Dyadic relationships are a major determinant of group-level social structure [17], and dyadic kinship influences partner choice and relationship strength [18]. As such, characterizing whether individuals have kin, non-kin or both available as social partners is a necessary step towards better understanding how social structure emerges from the kinship structure of groups. For example, a related group and a mix-related group that have the same mean relatedness might have different social structures: the related group might be rather cohesive, with many relationships of similar strength existing between group members, whereas the mix-related group might be more differentiated, with strong relationships existing between kin members and weak to no relationships between non-kin. Understanding how commonly individuals live with kin and/or non-kin can also help us further unravel the cost-benefit trade-off of group-living. For example, if most mammal groups are related, we might predict that indirect fitness benefits are key in offsetting the costs of group-living, whereas if most groups feature non-kin, we might predict that the benefits of group-living outweigh potential losses of indirect fitness. We focused on the kinship composition of same-sex group members because the kinship between female–male dyads is already the focus of a large body of work that indicates groups might feature unrelated female–male dyads owing to inbreeding avoidance and their interactions might be driven by reproduction alone [19].

Building on comparative studies of mean relatedness [11,13,14], we ask if unit size, dispersal patterns, number of breeding females and litter size predict kinship composition in mammals. Given the foundational nature of our study, we chose these socio-ecological traits because each of them is thought to influence and/or have been shown to correlate with mean relatedness [11,14]. We provide predictions in the methods for each of these traits and we test if they are related to our measure of kinship composition. We also examine the evolutionary trajectory of kinship composition in mammals. Because kinship composition data are still scarcely available in the literature, we present our dataset as a foundational dataset and our analyses as an initial exploration into what we can learn about kinship composition with current data and to discuss what we might be able to learn in the future. Accordingly, our interpretation of the results of the analyses reflects the preliminary nature of the dataset.

## 2. Methods

### 2.1. Key definitions and data collection

To ensure that our dataset was fully comparable, we started by proposing clear definitions for concepts (social unit, kinship composition, kin and non-kin) that could vary across studies. We then conducted a primary literature search and built a dataset in line with FAIR (findability, accessibility, interoperability, reuse) science principles [20]. The conclusions of comparative studies are only as good as the quality of the data they compare and the data's suitability to answer the proposed questions [21–26]. We therefore used inclusion criteria that ensured studies provided as conclusive information about kinship composition as possible.

#### 2.1.1. Social unit

We evaluated kinship composition at the level of the social unit. We defined a social unit (or unit) as the largest temporally stable social aggregation that includes two or more adult individuals of the same sex [27,28]. The term ‘group’ is often used to represent different types of social aggregations (e.g. temporally stable aggregations, individual-based fission–fusion aggregations), so to avoid confusion we use the term ‘social unit’ instead. We considered a social unit to be temporally stable if its composition outlasted any event that may temporarily affect it [28]. The simplest example of a social unit consists of a unit in which individuals form a long-term stable aggregation, wherein changes in membership are owing to death, birth or migration [28]. Among others, events that may temporarily affect the composition of a group include cyclical solitary dispersal during the mating season of members of an otherwise stable long-term aggregation; the formation of short-term subgroups within a larger, stable aggregation; or the temporary aggregation of a solitary individual to a stable aggregation. For example, in a population wherein females form temporally stable female-only aggregations but solitary males join the females for the mating season, we would consider that only the females live in a ‘social unit’ but males do not [28]. Our definition of social unit included group-based fission–fusion societies but excluded individual-based fission–fusion societies. Group-based fission–fusion societies are characterized by stable membership at the level of the social unit and changes to the composition of a unit represent death or migration events [28]. Our definition of social unit was also independent of the presence of immature individuals to avoid considering pre-dispersal parent–offspring dyads. For example, if a social unit consisted of multiple adult females, a sole adult male and multiple immatures of both sexes, we considered that only the adult females lived in a social unit for the purpose of our analyses, as there would be no same-sex adults for the single male to be related or unrelated to.

#### 2.1.2. Kinship composition

We quantified kinship composition for adult unit members of the same sex (although in one case data from immature offspring of both sexes helped to determine the kinship composition of the adult females of a unit, see the electronic supplementary material, S1). We classified same sex members of a unit into one of three possible kinship composition categories: (i) related, in which all same sex members are related; (ii) unrelated, in which all same sex members are unrelated; and (iii) mix-related, in which same sex members include related and unrelated individuals. Kinship composition classifications can therefore differ across sexes from the same unit; females of a social unit might be related, males unrelated.

The way we quantify kinship composition is not without its limitations. For example, if a very large unit is related and another large unit features a single unrelated dyad but is in everything else equal to the related unit, kinship composition would deem the two units as different when they are ecologically and socially nearly identical. In this situation, quantifying kinship composition on a continuous level by determining the proportion of kin (or non-kin) dyads present might be more useful. However, dyadic relatedness data are scarce and reported differently across sources, currently hindering our ability to build a comparative dataset of kinship composition on a continuous scale. In this first attempt to systematically characterize mammalian kinship composition, we used a categorical classification.

#### 2.1.3. Kin and non-kin

We classified dyads as kin if they were parent-offspring, grandparent-grandoffspring, or if they shared a recent ancestor, i.e. at least one of their parents or known grandparents were shared. If none of these criteria were met, we classified the dyad as non-kin. Kin-biases in interactions are usually limited to close kin in social mammals (e.g. *r* ≥ 0.125 [29]) so this two-generation criterion allowed us to use rare pedigree data while still being conservative enough that groupmates whose relatedness fell within this kin-bias threshold were probably considered kin. A two-generation criterion could result in a bias for related social units because it is easier to show that individuals share a recent ancestor than to obtain evidence that they do not. This criterion could also result in a bias for diurnal species, as it may be easier to obtain and maintain pedigrees for diurnal animals. Finally, our criterion could also result in a bias towards short-lived species, because two-generations of data are faster to generate for short-lived species than long-lived species. We expect this issue to be minimized, however, by the fact that several field sites on long-lived mammals maintain multigenerational data [30,31].

#### 2.1.4. Data collection

We conducted the following literature search on Webofknowledge: ‘(relatedness OR kinship OR nepotism) AND (pedigree OR coancestry OR genealogy) AND {mammal genus}’. The term {mammal genus} corresponded to all 1313 mammal genera (excluding *Homo*) as identified by [32]. We considered all papers returned by this literature search up until the 9 September 2022. We complemented this search by checking published datasets of mammal relatedness [11–14,33,34]. In line with the FAIR science principles [20], we did not consider data that were not publicly available. To establish kinship between dyads within social units, we used pedigree data [16] built from maternal genealogies and/or parentage analysis of genetic data. Although pedigree data are rare, we were unable to use more commonly available purely genetic dyadic data (e.g. pairwise estimations of kinship based on molecular markers) because these data suffer from high levels of dyadic kinship misclassification [15,16,35]. Contrary to pedigree data, purely genetic data alone cannot classify dyads as kin or non-kin, and thus cannot be used on their own to classify the kinship composition of groups, regardless of whether a categorical or continuous classification is used.

In particular circumstances, genetic relatedness data can be combined with demographic and/or incomplete kinship data to classify units as mix-related when using a categorical classification. When averaged over many dyads, estimates of dyadic relatedness using molecular markers usually correspond well with expected mean relatedness values derived from pedigrees [16], although a cluster of dyads that are on average positively related might still include unrelated dyads and vice-versa. If data suggest that the dyads in a positively related cluster are from the same breeding line and/or were born in the same unit, we can be confident that the cluster features at least some related dyads. Likewise, if data suggest that dyads from a zero/negatively related cluster are from different breeding lines and/or were born in different units, we can be confident that the cluster features unrelated dyads. As such, this allowed us to classify units as mix-related if they contained both types of clusters. For example, within a unit with various suspected matrilines from incomplete maternal genealogies and/or mitochondrial DNA, if mean relatedness between members of each matriline was positive and mean relatedness between members of different matrilines was zero/negative, we classed that unit as mix-related. We used genetic relatedness data to classify the kinship composition of units from three populations and to complement pedigree-only data in three other units (electronic supplementary material, S2).

We did not consider data from units whose mating behaviour and/or group composition was managed, e.g. captive units. The list of papers that we reviewed and the reasons why they were included or excluded can be found in the electronic supplementary material, S2. Papers that were not included in our dataset were those that: were not from a species/population of interest (e.g. captive units or solitary species); or if the data did not allow classification of kinship composition (e.g. because kinship composition data were estimated purely based on molecular markers; were not publicly available; or were not reported at the level of the social units). This could be the case even for studies that report pedigree data. For example, if the pedigree data were not from a unit of interest, were not reported in a way that allowed us to extract the kinship between all same-sex animals that lived concurrently in a unit, or were not publicly available. In other words, even well-known mammalian field sites with long-term pedigree data [30,31] may not appear in our dataset. The metadata for our kinship composition dataset can be found in the electronic supplementary material, S2.

### 2.2. Data analyses

To create a visual representation of categorical kinship composition in social mammals, we built a phylogenetic tree in the R environment [36]. We used a mammalian supertree [37] that has been used in previous comparative analyses [38]. We used the function ‘drop.tip’ from the package ‘ape’ [39] to truncate the tree to include only the species present in our dataset.

We assessed possible socio-ecological predictors and the evolutionary trajectory of kinship composition in mammals. We obtained information on unit size, dispersal patterns, number of breeding females and litter size from each paper in our dataset when available. When this information was not explicit, we searched other primary literature from the same units/populations from which we collected kinship composition data. The definitions we used for unit size, dispersal patterns, number of breeding females and litter size, along with details about how we collected these data, are available in the electronic supplementary material, S1. We predicted that larger unit size, dispersal, presence of multiple breeding females and monotocy (production of one offspring at a time) would increase the likelihood that units are mix-related or unrelated. Larger unit sizes tend to be associated with reduced relatedness between individuals [11,14] because large units are likely to be achieved by the presence of many breeding lines, the recruitment of unrelated immigrants and/or the merging of smaller units. Dispersal of individuals between units may also impact kinship composition by increasing the likelihood that units feature unrelated individuals [14]. The presence of multiple breeding females might dilute maternal relatedness between unit members [11,13,14], possibly leading to the establishment of matrilines that consist of related and unrelated unit members. Polytocy might be associated with kin living together because larger litters create cohorts of close maternal kin [14]. By contrast, monotocy might be associated with living with non-kin as it does not allow for maternal siblings to be born in the same cohort [14]. We note, however, that in monotocous species there is still the possibility that individuals born in the same cohort are related via paternal lines as offspring born from different females can still share the same father. We used the package ‘brms’ [40,41] to test for a correlation between the social predictors and kinship composition using Bayesian models. We built two models with kinship composition as the dependent variable. In one model, the predictor was mean adult female unit size, and in the other model the predictor was litter size. We were unable to test for a relationship between kinship composition and dispersal or number of breeding females because our sample exhibited little variation in these features. We set the models with a Bernoulli distribution, and controlled for phylogeny by including a covariance matrix of phylogenetic relatedness between the study species as a group-level effect [42]. We considered that there was a relationship between the predictor and the dependent variable when the credible interval (CI) of the posterior distribution of the predictor did not span 0, indicating that the estimated effect of the predictor is systematically different from 0.

We also used a Bayesian approach to conduct an initial assessment of the evolutionary trajectory of kinship composition in mammals. We used the function ‘make.simmap’ from ‘phytools’ to perform stochastic character mapping [43,44]. We used the phylogenetic tree we constructed before, set the number of simulations to 10 000 and used the ‘symmetrical-rates’ model of trait change. All analyses were conducted in R [36]. Owing to the preliminary nature of our dataset, we refrained from making strong inferences from the results. Full details about the statistical analyses are available in the electronic supplementary material, S1. The code used to perform the analyses is available in the supplementary Code file.

## 3. Results

Our literature search resulted in 331 papers, 211 of which were from the Webofknowledge search. Kinship composition data that matched our criteria and definitions were found in 22 of those publications (table 1). Kinship composition data were available for 18 species, comprising 76 datapoints (table 1). Datapoints comprise the same unit in different periods, amounting to a total of 67 female datapoints from 62 units of 21 populations of 17 species, and 11 male datapoints from 11 units of five populations of five species.

**Table 1.** Kinship composition, number of populations, units and datapoints, mean unit size, dispersal and philopatry patterns, number of breeding females and litter size of our sample of species. (Data are reported as a single datapoint per sex/species. Mean unit size was calculated by averaging unit sizes from all single datapoints. Numbers in square brackets [] refer to the references from which we extracted the respective data. The metadata of the data presented in this table can be found in the electronic supplementary material, S2.)

species	kinship composition	sex	no. of populations, no. of units, (no. of datapoints)^a^	mean unit size (mean ± s.d.)	dispersal/philopatry	no. breeding females	litter size
*Cryptomys damarensis*	related [45]	F	3,15 (15) [45]	6.2 ± 2.8 [45]	philopatric [45]	singular breeding [45]	polytocous [46]
*Ctenodactylus gundi*	related [47]	F	1,2 (2) [47]	3.0 ± 1.4 [47]	philopatric [47]	plural breeding [47,48]	polytocous [48]
	related [47]	M	1,2 (2) [47]	2.5 ± 0.7 [47]	philopatric [47]	–	–
*Marmota flaviventris*	related [49]	F	2,2 (2) [49]	2.5 ± 0.7 [49]	philopatric [50]	plural breeding^b^ [49]	polytocous [51]
*Cynomys ludovicianus*	related [52]	F	1,3 (3) [52]	2 [52]	philopatric [52,53]	plural breeding [52]	polytocous [54]
*Macaca mulatta*	mix-related [55,56]	F	1,5 (9) [55,56]	53.1 ± 23.8 [56,57]	philopatric [55,56]	plural breeding [55,56]	monotocous [58]
*Macaca fascicularis*	mix-related [59]	F	1,1 (1) [59]	13 [60]	philopatric [59]	plural breeding [60]	monotocous [60]
*Papio cynocephalus*	mix-related [61,62]	F	1,3 (3) [62]	9.7 ± 1.2 [62]	philopatric [61]	plural breeding [61,62]	monotocous [63]
*Cercopithecus mitis*	mix-related [64]	F	1,3 (3) [64]	15.2 ± 5.2 [64]	philopatric [64]	plural breeding [64]	monotocous [65]
*Pan troglodytes*	mix-related [66]	F	1,1 (1) [66]	39 [66]	disperse [66]	plural breeding [67]	monotocous [68]
	mix-related [67]	M	1,1 (1) [67]	12 [67]	philopatric [67]	–	–
*Cebus capucinus*	related [69]	F	1,2 (2) [69]	3.0 ± 1.4 [69]	philopatric [69]	plural breeding [69]	monotocous [70]
*Myotis bechsteinii*	mix-related [71]	F	1,4 (4) [71]	22.0 ± 9.0 [71]	philopatric [71]	plural breeding [71]	monotocous [72]
*Eptesicus fuscus*	mix-related [73]	F	1,1 (1) [73]	37.5 [73]	philopatric^c^ [73]	plural breeding [73]	monotocous^d^ [74]
*Artibeus jamaicensis*	related [75]	M	1,2 (2) [75]	2.0 ± 0.0 [75]	unclear [75]	plural breeding [75]	monotocous [76]
*Orcinus orca*	related [77]	F	1,12 (12) [78]	3.2 ± 1.2 [78]	philopatric [77]	plural breeding [77]	monotocous [77]
	related [77]	M	1,5 (5) [78]	2.4 ± 0.5 [78]	philopatric [77]	–	–
*Canis lupus*	related [79,80]	F [79,80]	2,3 (4) [79,80]	2.0 ± 0.0 [79,80]	philopatric [79]	singular breeding [79]	polytocous [79]
	related [80]	M [80]	1,1 (1) [80]	3 [80]	unclear	–	–
*Cuon alpinus*	related [81]	F	1,1 (1) [81]	2 [81]	disperse [81]	singular breeding [81]	polytocous [81]
*Vulpes vulpes*	related [82]	F	1,1 (1) [82]	5 [82]	philopatric [82]	plural breeding^e^ [82]	polytocous [82]
*Crocuta crocuta*	mix-related [83]	F	1,1 (1) [83]	21 [84]	philopatric [83]	plural breeding [83]	monotocous [85]

^a^Not all kinship data and/or unit size data from all sources met our inclusion criteria. The data reported in this table are the data that met our criteria. See Data Collection sections above and in the electronic supplementary material, S1; as well as the kinship composition metadata in the electronic supplementary material, S2 for more information.

^b^*Marmota flaviventris*’ female social units consist of the kin groups. Given our definition of social unit, we focus on the kin groups with two or more adult females. Most studies focus on the male social unit, which consists of harems of one or more female kin groups. Yet, Armitage & Johns [49] appear to suggest that when there is more than one adult female in a kin group, all can/will reproduce.

^c^Only 10–30% of immature females return to their natal roost owing to unclear reasons, which could be low winter survival, dispersal of juveniles, etc. Yet, adult females are strongly philopatric [73]. Given the uncertainty associated with the patterns of movement of immature females, we used adult female movement as a proxy.

^d^Litter size of *Eptesicus fuscus* varies between a litter of one in western North America and Caribbean and of two in eastern North America. The litter size for the population from which we extracted kinship composition data (Cypress Hill, Canada [73]) is not clearly reported. Neubaum *et al*. [74] examined litter size in two maternity roosts that contained west and east lineages and found that litter size is probably a function of geography, and thus of population density and available resources. Their results, however, indicated that 86% of litters contained only one offspring across both roosts, 75% in one of the roosts and 47% in the other. Given the lack of more specific data, we classified *E. fuscus* as monotocous.

^e^Number of breeding females for the population of *Vulpes vulpes* is highly variable and appears to be connected with the density of the population [82]. Both in high and low densities, most units appear to feature a single breeding female [82], but the unit for which we were able to collect kinship composition and unit size data featured more than one breeding female. The unit for which we collect kinship composition and unit size data was unit A in 2003 (fig. 2 of [82]) as we could not conclusively match kinship composition and unit size data for any other units given the information in table 1 of [82].

In total, our sample represented 18.52% of all extant mammalian orders. The species in our sample were all members of the magnorder Boreoeutheria, which includes the superorders Laurasiatheria and Euarchontoglires (electronic supplementary material, S1: figure S2). Our sample represented two out of five extant orders that make up the Laurasiatheria and three of the six Euarchontoglires orders and included Primata (*n* = 6), Carnivora (*n* = 4), Rodentia (*n* = 4), Chiroptera (*n* = 3) and Artiodactyla (*n* = 1) [32]. These orders are five of the six most speciose orders, accounting for 80.86% of all extant mammal species (5252 out of 6495) according to the latest version of the Mammalian Diversity Dataset [86].

**Figure 1.**
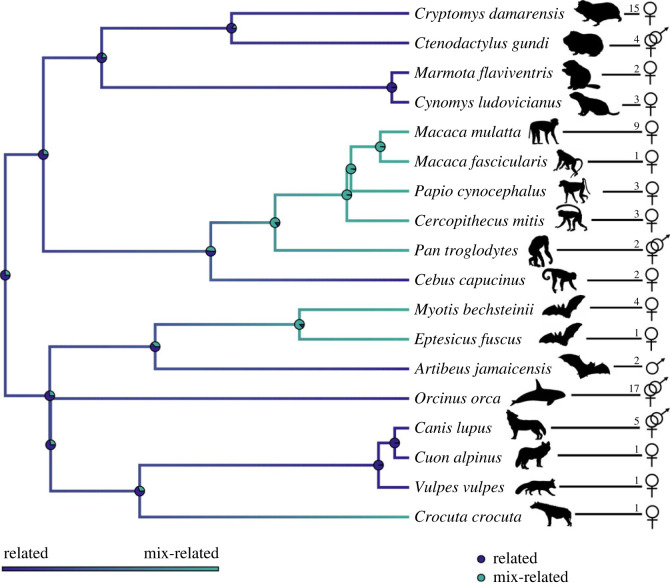
Kinship composition in mammals. We identified 10 species that had related units (dark blue on the tips of the tree) and eight species that had mix-related units (teal on the tips of the tree). Numbers next to the species names represent the number of datapoints for each species. The symbols ♀ and ♂ indicate whether the data refer to the kinship composition of females or males, respectively. The phylogenetic tree represents the evolutionary relationships of our sample of species. Branch and pie chart colours are a visual representation of the stochastic character mapping's posterior probability of the states of kinship composition across all edges and internal nodes of the tree, wherein related is dark blue and mix-related is teal. This visual representation is based on the stochastic character mapping analysis, which reflects the preliminary nature of the data, so should not be taken as a conclusive result. See the electronic supplementary material, S1: figure S2 for a visual representation of these data on a tree with all mammalian orders. The silhouette referent to *Macaca mulatta* was provided by Delphine De Moor and those referent to *Cuon alpinus* and *Orcinus orca* were provided by Melissa A. Pavez-Fox. The silhouette referent to *Ctenodactylus gundi* (credit: Flappiefh) was taken from https://commons.wikimedia.org/ and those referent to *Cebus capucinus* (credit: Sarah Werning) and *Artibeus jamaicensis* (credit: Roberto Díaz Sibaja) were taken from http://phylopic.org/. These three figures were available under the Creative Commons Attribution 3.0 Unported licence (https://creativecommons.org/licenses/by/3.0/). All other animal silhouettes were available under the Public Domain Dedication 1.0 licence or the Public Domain Mark 1.0 licence from http://phylopic.org/.

Ten of the 18 species in our sample were found to have related units and eight species had mix-related units (table 1; figure 1). Females from nine species and males from four species were related, and females from eight species and males from one species were mix-related (table 1; figure 1). No species in our sample had fully unrelated units. When data for more than one unit of the same species were available, units had the same kinship composition in all cases (*n*_species_ = 13). When female and male data were available for the same species, we found females and males to be of the same kinship composition (*n*_related_ = 3, *n*_mix-related_ = 1). All orders for which we have data for more than one species featured units with different kinship composition, except for Rodentia, which featured only related units (figure 1).

Our analysis of the predictors of kinship composition suggested that unit size predicts kinship composition in the females, with smaller units being more likely to be related and larger units to be mix-related (effect of unit size on kinship composition state: estimate: 6.6; 95% CI: 1.0, 17.1; mean unit size ± standard deviation (s.d.) for related units: 3.2 ± 1.5; mean unit size ± s.d. for mix-related units: 26.3 ± 15.2). In our sample, females were philopatric (*n*_related_ = 8, *n*_mix-related_ = 7), except for two species in which females dispersed. One of the female-dispersing units was classed as related and the other mix-related. Males in our sample were philopatric too (*n*_related_ = 2, *n*_mix-related_ = 1), except for two species in which the dispersal patterns of males was unclear. Both those units were classed as related. Only three species were singular breeders, all of which formed related units (table 1). In our sample, 11 species were monotocous, of which three formed related units and eight formed mix-related. The other seven species were polytocous, all of which formed related units (table 1). We found no evidence that litter size predicts the kinship composition of females (effect of litter size on kinship composition state: estimate: −9.0; 95% CI: −24.2, 9.0). Stochastic character maps suggested a convoluted path in the evolution of kinship composition, with an average of 18.3 state changes (electronic supplementary material, S1: figure S1). The average number of state changes spanned the sample size of our dataset, suggesting that the dataset does not span enough species to robustly infer the evolutionary trajectory of kinship composition in mammals. A full description of the results of the stochastic character mapping analysis is available in the electronic supplementary material, S1.

## 4. Discussion

Here, we provided an initial quantification of the same-sex kinship composition of social mammals. Out of a sample of 18 species, we found that 10 species lived in related units and eight in mix-related units. Our results suggest that living with unrelated individuals may not be rare in mammal societies. Our results are in line with those of Lukas *et al*. [11] and Lukas & Clutton-Brock [13], who found evidence that many mammals live in units with low mean relatedness. By categorising kinship structure using dyadic kinship composition, we were able to expand on those results and explicitly conclude if a unit included unrelated members or not, which is not always clear from mean relatedness. For example, female rhesus macaques (mean *r* = 0.15) and capuchin monkeys (mean *r* = 0.19) were reported to have similar mean relatedness in [13], but rhesus macaque females live in mix-related units [55,56], while female capuchin units are fully related [69]. Additionally, while our literature search did not return unit-level female Kalahari meerkat (*Suricata suricatta*) data that fitted our criteria (see below why this was the case), it is possible that female meerkats sometimes associate with unrelated females [87] despite having high mean relatedness (*r* = 0.42; [13]). Future reporting of the kinship composition of meerkat social units might clarify this possibility, and would be a clear example of a species whose units have a very high mean relatedness but feature non-kin. Lukas & Clutton-Brock [13] found that mean relatedness predicted the type of social complexity exhibited by females mammals, but kinship composition might reveal divergent patterns. Future work on socio-ecological dimensions, such as on the evolution of different care systems and social structures, might benefit from the coupled use of kinship composition data and mean relatedness data.

Kinship composition is also a necessary concept to better understand the evolution of cooperation and how groups are formed and maintained. All units in our sample featured at least some close relatives, suggesting that there is a clear opportunity for within-unit cooperation to offset some of the costs of group-living in social mammals via indirect fitness benefits. This is in line with relatedness being a key determinant of the emergence of group-living and the strength of cooperation across animal societies [4]. However, our sample also indicates that a variety of mammal species have the opportunity to cooperate with non-kin, suggesting that, in some cases, direct benefits play an important role in offsetting the costs of group-living and in the maintenance of cooperation [4,88]. We did not find unrelated units, possibly because of the two-generation criterion we used to classify dyads as related or unrelated. Unrelated units might arise as the result of migrants from various independent units grouping together. Establishing kinship between individuals from independent units might require deep pedigree data, which is hard to obtain and may not yet be available for most field sites. As such, it might be difficult to identify unrelated units. Future population-level pedigree data from long-term field sites could reveal the existence of unrelated units and, thus, of mammal societies where kinship does not play a role in the formation and maintenance of groups.

Living with non-kin does not necessarily imply that non-kin cooperate. For example, living with kin and non-kin might be a result of selection for larger group sizes that cannot be achieved by living with kin alone and it is possible that interactions between unrelated individuals are solely competitive. The potential for indirect fitness benefits from kin might reduce the prevalence of positive interactions between unrelated individuals. This can result in differentiated relationships, culminating in a general pattern of kin-based social modularity. In fact, members of groups in which mean relatedness is high are more likely to cooperate indiscriminately, whereas members of groups in which mean relatedness is low are more likely to direct cooperative behaviour to specific mates [13]. Still, there is evidence of affiliation between non-kin or distantly related individuals in seven out of eight mix-related species in our sample [61,64,66,73,89–94]. Given that affiliation between non-kin appears to be widespread across mix-related units, it is important to understand why non-kin cooperate, even when individuals have the opportunity to limit their interactions to kin.

Non-kin might constitute valuable partners under different conditions. When individuals have a limited number of close kin available, they might extend their network to non-kin. Individuals might form a non-kin ‘safety network’ from which they can gain the benefits of social interactions when kin are socially unavailable or absent (e.g. because they died) as a social bet-hedging strategy [95], or when they need more resources than what kin alone can provide [96]. Similarly, occasional interactions with non-kin might be a useful way to obtain benefits that kin might not be able to provide [9,93], such as information about the environment. Additionally, individuals might prioritise interacting with non-kin, even at the expense of kin, if unrelated partners provide direct benefits that outweigh kin-based inclusive fitness benefits [88]. For example, non-kin might be preferred over kin if non-kin are more competent at providing rare or highly valuable commodities, such as coalitionary support [88,97,98]. Additionally, under strong competition between units, increased within-unit cooperation with kin and non-kin alike might be beneficial [99]. Individuals might also interact with non-kin if they are not able to discriminate kin from non-kin, although this is unlikely as social mammals have been shown to be able to discriminate kin from non-kin [100–102].

Perhaps surprisingly, populations with multi-generational pedigrees, e.g. Mweya's banded mongooses (*Mungos mungos*) [103,104], Kalahari's meerkats [105], Isle of Rum's red deer (*Cervus elaphus*) [106], St Kilda's Soay sheep (*Ovis aries*) [107]; and species with known relatedness data, e.g. *Octodon degus* [108,109], *Rhombomys opimus* [110], did not feature in our dataset. The existence of relatedness data does not mean that the data are openly available or reported in a way that the kinship composition of units can be extracted. As a specific example, our literature search returned several studies from the Kalahari meerkat population (electronic supplementary material, S2). Yet we could not use the high quality pedigree data in these papers. The pedigree data were sometimes reported across the sexes and others at the population level, neither of which allowed us to ascertain the kinship composition of specific units. Recently, Makuya *et al*. [111] highlighted that social organization data are often not fully or clearly reported in the published literature, ultimately hampering advances in our understanding of the evolution of sociality. For kinship composition specifically, it is important that, when possible, future studies report their pedigree data together with demographic data, such as the identity, age and sex of members of the same social unit, in line with the FAIR science principles [20,111]. Comparative research crucially relies on data shared in an open and standardized way [20,111]. It is also noteworthy that some species with known relatedness data did not clearly fit our definition of a social unit, e.g. female bottlenose dolphins (*Tursiops truncatus*) [112] and red squirrels (*Tamiasciurus hudsonicus*) [113].

We found that, in our dataset, larger units were more likely to be mix-related and smaller units were more likely to be related. It is possible that mix-related units arise as a by-product of selection for larger unit size and the kinship composition of mammal groups might partly be driven by group size. Species in mix-related units may be under selective pressure to live in larger units owing to predation risk or competition for resources between units to defend territories and/or food resources [2,3,114,115]. However, we should note that using a binary measure of kinship composition could also drive this result purely by chance—as unit size increases, the probability that any two individuals are unrelated increases. Confirming this preliminary finding with continuous kinship composition data will be important to come to grips with any relationship between group size and the tendency for mammals to live with non-kin. Litter size did not predict kinship composition. However, all mix-related species were monotocous and plural breeders, suggesting that monotocy facilitates the emergence and/or maintenance of mix-relatedness, possibly by facilitating the emergence/maintenance of independent kin-lines [14,38]. All polytocous species were related, which is in line with polytocy facilitating the emergence of units with high relatedness [14,38]. It is important to note that offspring from different mothers can still be related via paternal lines. Male reproductive skew and tenure could be included as possible socio-ecological predictors in future analyses as both high male reproductive skew and long tenure would be predicted to lead to (paternal) kin living together. As pedigrees get deeper, future kinship composition data will allow for a closer investigation of whether groups consist of maternal kin, paternal kin or both. The results of the stochastic character mapping suggest that our dataset does not span enough species to robustly infer the evolutionary trajectory of kinship composition in mammals.

It is important that our dataset is expanded upon by future research and our results revisited as new kinship data become available. The addition of new kinship composition data to our dataset could provide new clues regarding the socio-ecological predictors and evolutionary trajectory of kinship composition that our initial analyses could not. Once the relationship between kinship composition and its socio-ecological predictors is clarified, this might open up the possibility to use socio-ecological factors, such as dispersal patterns and number of breeding females, as proxies of kinship composition to answer questions that require larger datasets. Crucially, the use of such proxies requires them to first be validated by a dataset based on strict criteria, for which the definitions and initial dataset set forth in this paper provide the foundation. It is also important that future kinship composition data from under-represented taxa are added, as the species in our dataset were all members of the magnorder Boreoeutheria [32] (electronic supplementary material, S1: figure S2), suggesting that long-term studies might be biased towards these types of mammals. Data from the magnorder Atlantogenata would allow for a preliminary reconstruction of the evolution of kinship composition to the last common ancestor of placental mammals. The addition of new data will also probably reveal within-species variation in kinship composition, as within-species variation in mean relatedness [116] and social organization [26] is ubiquitous. As our understanding of kinship composition increases, including how it varies over time and across the life of individuals, so will our capacity to answer other important questions about the evolution of animal societies [117]. Such data might help better understand the evolution of life-history traits like menopause [116,118] and how age-dependent variation in relative reproductive potential might influence the value of kin as sources of indirect fitness benefits [119].

Here, we provided a first overview of the taxonomic representation of kinship composition in social mammals by assembling an initial kinship composition dataset. Contrary to what may have been expected, our results suggest that living with a mixture of related and unrelated individuals is far from rare in social mammals. This indicates that indirect and direct fitness benefits have probably worked in concert to help offset the costs of group-living and promote within-unit cooperation throughout the evolutionary trajectory of mammalian societies. We are hopeful that the dataset presented here will encourage the scientific community to report existing and future kinship composition data, thus opening new avenues in the study of the evolution of sociality.

## Data Availability

The data, metadata and code are provided in the main text and the electronic supplementary material [120].
